# YKL-40 tissue expression and plasma levels in patients with ovarian cancer

**DOI:** 10.1186/1471-2407-9-8

**Published:** 2009-01-09

**Authors:** Estrid VS Høgdall, Merete Ringsholt, Claus K Høgdall, Ib Jarle Christensen, Julia S Johansen, Susanne K Kjaer, Jan Blaakaer, Lene Ostenfeld-Møller, Paul A Price, Lise H Christensen

**Affiliations:** 1 Department of Virus, Hormones and Cancer, Institute of Cancer Epidemiology, Danish Cancer Society, Copenhagen, Denmark; 2 Department of Pathology, Herlev Hospital, University of Copenhagen, Copenhagen, Denmark; 3 Faculty of Medical Laboratory Science, University College Oeresund, Copenhagen, Denmark; 4 The Gynaecologic Clinic, The Juliane Marie Centre, Rigshospitalet, University of Copenhagen, Copenhagen, Denmark; 5 Finsen Laboratory, Rigshospitalet, Copenhagen Biocenter, University of Copenhagen, Copenhagen, Denmark; 6 Department of Rheumatology, Herlev Hospital, University of Copenhagen, Denmark; 7 Department of Gynecology and Obstetrics, Aarhus University Hospital, Skejby, Aarhus, Denmark; 8 Department of Biology, University of California, San Diego, La Jolla, CA, USA; 9 Department of Pathology, Bispebjerg Hospital, University of Copenhagen, Copenhagen, Denmark

## Abstract

**Background:**

YKL-40 (chitinase-3-like-1) is a member of "mammalian chitinase-like proteins". The protein is expressed in many types of cancer cells and the highest plasma YKL-40 levels have been found in patients with metastatic disease, short recurrence/progression-free intervals, and short overall survival. The aim of the study was to determine the expression of YKL-40 in tumor tissue and plasma in patients with borderline ovarian tumor or epithelial ovarian cancer (OC), and investigate prognostic value of this marker.

**Methods:**

YKL-40 protein expression was determined by immunohistochemistry in tissue arrays from 181 borderline tumors and 473 OC. Plasma YKL-40 was determined by ELISA in preoperative samples from 19 patients with borderline tumor and 76 OC patients.

**Results:**

YKL-40 protein expression was found in cancer cells, tumor associated macrophages, neutrophils and mast cells. The tumor cell expression was higher in OC than in borderline tumors (p = 0.001), and associated with FIGO stage (p < 0.0001) and histological subtype (p = 0.0009). Positive YKL-40 expression (≥ 5% staining) was not associated with reduced survival. Plasma YKL-40 was also higher in patients with OC than in patients with borderline tumors (p < 0.0001), and it was positively correlated to serum CA-125 (p < 0.0001) and FIGO stage (p = 0.0001). Univariate Cox analysis of plasma YKL-40 showed association with overall survival (p < 0.0001). Multivariate Cox analysis, including plasma YKL-40, serum CA125, FIGO stage, age and radicality after primary surgery as variables, showed that elevated plasma YKL-40 was associated with a shorter survival (HR = 2.13, 95% CI: 1.40–3.25, p = 0.0004).

**Conclusion:**

YKL-40 in OC tissue and plasma are related to stage and histology, but only plasma YKL-40 is a prognostic biomarker in patients with OC.

## Background

YKL-40 (chitinase-3-like-1) is a highly conserved protein [1] and a member of "mammalian chitinase-like proteins" [1,2]. The protein is expressed in many types of cancer cells (*dbest *NCBI database), and elevated plasma levels are predictive of poor prognosis in patients with different types of cancer [2-8]. The highest plasma YKL-40 levels have been found in patients with metastatic disease, short recurrence/progression-free intervals, and short overall survival [2-8]. Furthermore, plasma YKL-40 has provided independent information on prognosis over clinical characteristics and biomarkers, such as serum CA-125, LDH, PSA, CEA, and HER2 [2-8]. It has been suggested that YKL-40 is associated with cancer cell proliferation, differentiation, metastatic potential, and extracellular tissue remodelling, but *in vivo *proofs of these are yet to be obtained [2].

Immunohistochemical studies have demonstrated a strong cellular expression of YKL-40 in all germ layers of human embryos and fetuses, including ecto-, meso- and endoderm [9]. The expression is particular high in tissues characterized by rapid proliferation and marked differentiation, and in tissues undergoing morphogenetic changes [9]. A similar pattern is seen in normal adult human tissue, where YKL-40 is highly expressed in cells with a high cellular activity [10].

Neoplastic tissue has also been found to show a higher expression for YKL-40 than the normal counterpart [2]. In glioblastoma, YKL-40 protein expression has proven to be a biomarker of histologic subtypes [11], with high gene- and protein expression being associated with poor radiation response and early disease progression and death [11-13]. In addition, at recurrence YKL-40 is up-regulated in the tumor tissue [14] and elevated in serum [15].

High YKL-40 protein expression has also been found in carcinoma cells from breast [16-18], colon [2], liver [19], cervix [2], and head/neck [20], and skin (melanoma) [2], as well as within tumor-associated macrophages, mast cells and leukocytes [10,18,20-22]. In a small study of patients with breast cancer high YKL-40 protein expression in the cancer cells was associated with short disease-free survival [16]. However, this could not be confirmed in a recent large study of YKL-40 protein expression in biopsies from 630 patients with primary breast cancer; in this study there was no association between YKL-40 protein expression in the breast cancer cells and disease free survival and overall survival [18].

There are no published studies on YKL-40 protein expression in OC tissue. Preoperative serum or plasma concentrations of YKL-40 are elevated in 65% of patients with FIGO stage I and II, and in 74% to 91% of patients with FIGO stage III and IV [23,24]. Patients with early-stage OC [24], stage III [23], or recurrence of OC [25] and high plasma or serum levels of YKL-40 have shorter survival compared to patients with normal levels of YKL-40. No close association between serum YKL-40 and serum CA-125 and CA15-3 in patients with OC has been published [23-26]. In patients with FIGO stage III multivariate Cox analysis including preoperative plasma YKL-40, serum CA125, optimal vs. suboptimal primary surgery, age, and histological type of tumor, demonstrated that high plasma YKL-40 was an independent biomarker of short survival [23]. Furthermore, serum YKL-40 was a predictor of chemoresistance in the second-line treatment of OC patients [26].

The aim of this study was to determine YKL-40 tissue expression in borderline and epithelial OC tissues, its possible correlation to clinical-pathological parameters and plasma YKL-40 levels, and the prognostic value of both YKL-40 tissue expression and plasma levels.

## Methods

### Patient Population

The MALOVA study ("MALignant OVArian cancer study") is a multidisciplinary Danish study covering epidemiology, lifestyle factors, biochemistry and molecular biology with the purpose of identifying risk factors and prognostic factors for OC. The design of the MALOVA study is described in details elsewhere [27]. Briefly, preoperative blood samples and tumor tissue samples were obtained from the majority of the 681 OC patients and from the 235 women with borderline ovarian tumor were included in the MALOVA study. FIGO stages were obtained from clinical records and reviewed by two gynecologists specialized in OC. Patients were either classified as radically operated with no macroscopic residual tumor or non-radically operated with macroscopic residual tumor after surgery. On the retrospectively collected paraffin embedded tissues, histologic grading were performed individually by two persons.

### Follow-up

In Denmark all inhabitants have a unique personal (10-digit) identification number (CPR number), which is used universally in the Danish society. These identification numbers, which comprise information on date of birth and sex, are registered in the computerized Danish National Central Population Register. The register contains information on e.g. dates of death and emigration. All cases in this study were traced in the register and date of death, emigration or August 23^rd^, 2007, whichever came first, were registered. The relevant hospital files were collected and scrutinized and information on treatment (surgery and chemotherapy), was established. At the end of follow-up, a total of 490 OC patients had died (median follow-up time: 23 months, range: 1–111), and 191 OC patients were still alive (median follow-up time: 91 months, range: 66–121).

### Tissue array

The paraffin embedded tissue from the tumors was used for tissue array analyses. Forty-eight OC and 24 borderline ovarian tumors were excluded because of poor tissue quality or lack of tumor tissue in the collected blocks, and 132 OC and 16 borderline ovarian tumors were excluded due to unreadable YKL-40 expression results, lack of tumor cells in the selected TA cylinders, loss of tissue during the staining procedure or folding/tearing of sections by the microtome. Finally, 42 non-epithelial tumors (28 OC and 14 borderline ovarian tumors) were excluded because epithelial and non-epithelial ovarian tumors differ in embryologic and pathologic characteristics. Together, these exclusions left 473 OC and 181 borderline ovarian tumors, which were suitable for the YKL-40 tissue expression and survival analyses.

For the tissue array production, one to two representative areas were marked on a freshly cut H&E stained section from each selected block. Tissue cylinders with a diameter of 2 mm were punched from corresponding areas in the donor tumor block and brought into 2–4 individual recipient paraffin blocks (tissue array blocks), using a custom-made manual Tissue Array Instrument (Beecher Instruments, Silver Spring, MD, USA). Once the cores were laid into the recipient block, the paraffin was slightly melted in order to bind the cores into the block. This treatment secured the cores during sectioning. Melting was achieved by placing the blocks in an oven at 37°C for 10 min.

A total of 2 to 4 tissue arrays consisting of corresponding viable and representative tissues from each ovarian tumor were constructed in order to minimize the risk of intra-tumor variability. The tissue arrays were sorted with respect to histological subtype and FIGO stage. Four cores of control tissue (2 from kidney, 2 from liver) were placed strategically in each tissue array to ensure unique orientation of every tissue array together with a maximum of 31 different patient tumor samples.

### Tissue preparation and immunostaining of YKL-40

Two μm sections (Section Transfer System, STS, Ergostar HM200, MICROM International GmbH, Walldorf, Germany) from the tissue array blocks were transferred to glass slides (DAKO Chem-Mate Capillary Cap Microscope Slides, 57 mm, DAKO A/S, Glostrup, Denmark). Slides were stored at 4°C for a maximum of 8 days until staining for YKL-40.

Prior to staining, the sections were deparaffinized in xylene and rehydrated in graded dilutions of alcohol. In order to demask antigens within the tissue, sections were pretreated in TEG buffer, pH 9.0 (Tris 10 mmol/l and EGTA 0.5 mmol/l), followed by heating in a microwave oven to 98°C for 15 min. Sections were then left to cool in the buffer at room temperature for an additional 15 min. To block endogen peroxidase activity the sections were treated with 0.03% w/v hydrogen peroxide for 5 min. To avoid background staining the sections were then pre-incubated with 5% w/v purified bovine serum albumin (BSA) (Dade Behring, Liederbach, Germany) diluted in TRIS buffer pH 7.6 for 10 min. The primary specific mouse monoclonal antibody against human YKL-40 (201.F9, 3.8 mg/ml, isotype IgG2b, κ, epitope GAWRGTTGHHS, corresponding to the amino acids 210–220) was diluted 1:100 (38 ng/ml) in 1% w/v purified BSA (Dade Behring) in TRIS buffer pH 7.6 and incubated for 60 min. The secondary antibody EnVisionTM+System-HRP (DAB) (Dako, Glostrup, Denmark, Code K 400711-2, lot no. 104.225) was used for 30 min. The colour was developed with DAB added with chromogen for 10 min. Sections were rinsed in water, counterstained in Mayers Hematoxylin for 3 min and finally dehydrated, mounted and coverslipped with Pertex. Washings with 5 mM Tris buffer, pH 7.6 with NaCl 0.9% w/v and Tween 0.1% v/v (TBS) were used between all steps in the procedure. After heating all steps were performed at room temperature in a humidity chamber to avoid air-drying of the sections. The staining process was done manually.

### YKL-40 scoring of tissue expression

Two observers, both experienced in evaluating immunohistochemical stained tissues, simultaneously assessed the patterns of YKL-40 protein expression of each tumor sample. The observers had no knowledge of clinical parameters and study endpoint. Standardization of scoring was achieved by comparison of the scores, and any discrepancies were resolved by consensus. Scoring for YKL-40 protein expression was based on the proportion of cells in a given tumor specimen exhibiting distinct cytoplasmic immunopositivity as well as the intensity of staining (percentage scale: 0, 5, 10, 20, 30, 40, 50, 60, 70, 80, 90, and 100). Secondly, the YKL-40 scoring results were transformed into two scales: a two-tiered scale (1: negative; 2: ≥ 5% positive tumor cells) and a four-tiered scale (1: negative; 2: 5% positive tumor cells; 3: 10% positive tumor cells; 4: ≥ 20% positive tumor cells). The four tissue control cores of kidney and liver, respectively, showed consistent staining results. For the general description and the prognostic evaluation both scales were used.

### Plasma YKL-40 analysis

Blood samples were obtained no earlier than two weeks prior to surgery from the 95 patients operated at Herlev Hospital and Rigshospitalet, University of Copenhagen. EDTA-blood samples were left on the blood cells at room temperature for less than 8 hours. Following the samples were separated by centrifugation at 2000 g for 10 min. at room temperature and plasma EDTA samples stored in aliquots at -80°C until analysis. EDTA plasma samples collected from patients included in the MALOVA study, who had been treated at other hospitals were not used for plasma YKL-40 measurements, since the plasma obtained from these centers had not been separated from the blood cells within 8 hours after sampling. It is known that EDTA plasma YKL-40 is only stable when samples are processed within 8 hours [28].

Plasma levels of YKL-40 were determined in duplicates by a commercial two-site, sandwich-type enzyme-linked immunosorbent assay (Quidel, Santa Clara, CA, USA) using streptavidin coated microplate wells, a biotinylated-Fab monoclonal capture antibody, and an alkaline phosphatase-labeled polyclonal detection antibody. The detection limit was 20 μg/l. The intra-assay coefficient of variation (CV) was ≤ 5.0% and inter-assay CVs ≤ 10.2%. The samples were analyzed blinded to clinical parameters and study endpoint.

### Healthy subjects

The reference interval for plasma YKL-40 was determined in 144 healthy women (median age 51 years, range 18–79 years) characterized by not being on medication and having no signs of pre-existing disorders such as joint, liver, metabolic or endocrine disease or malignancy.

### Ethics

Written informed consent was obtained from all patients. The study has been approved by the scientific ethical committee in the study area (KF01-384/95).

### Statistical analyses

Statistical comparisons between groups were carried out using Chi-square or rank sum tests. Association between YKL-40 tissue expression levels and plasma YKL-40 levels was assessed using the Spearman correlation. The clinical endpoint in the OC patients was survival determined as the time from baseline blood sample before operation to time of death updated at August 23^rd^, 2007. Patients who died from non-related OC were censored in the survival analyses at the date of death. Cases in which patients were alive by this date were censored. Survival probabilities were estimated by the Kaplan-Meier method and tests for differences between strata were done using the log-rank statistic. Graphical presentation using Kaplan-Meier estimates of survival were shown grouping the patients by YKL-40 expression in the tumor tissue dichotomized at 5%. Graphical presentation using Kaplan-Meier estimates of survival were shown grouping the patients by plasma YKL-40 levels dichotomized as normal or elevated compared to age-matched controls. In the univariate survival analyses the YKL-40 expression scale in tumor tissue was dichotomized as < 5% vs. ≥ 5% and the plasma YKL-40 levels by the actual value on the log scale (natural) (log transformed). The multivariate Cox regression multivariate analysis was based on FIGO stage, residual tumor after primary surgery, serum CA-125, age and plasma YKL-40 levels by the actual value on the log scale (natural) (log transformed). Confidence intervals (95% CI) were based on Wald's test statistic for the corresponding parameters in the Cox regression model, i.e. on the log-scale for the hazard ratios (HR). Model assessment was done using graphical methods, Schoenfeld and martingale residuals. P-values less than 5% were considered significant. All calculations were performed using SAS (version 9.1, SAS Institute, Cary, NC, USA).

## Results

### YKL-40 protein expression in borderline ovarian tumors and in ovarian cancer tissue

Tissue arrays for YKL-40 immunohistochemical analysis were available from 181 patients with borderline ovarian tumors (median age 54 years, range 33 – 80) and from 473 patients with OC (median age 59 years, range 26 – 80).

**Figure 1:**
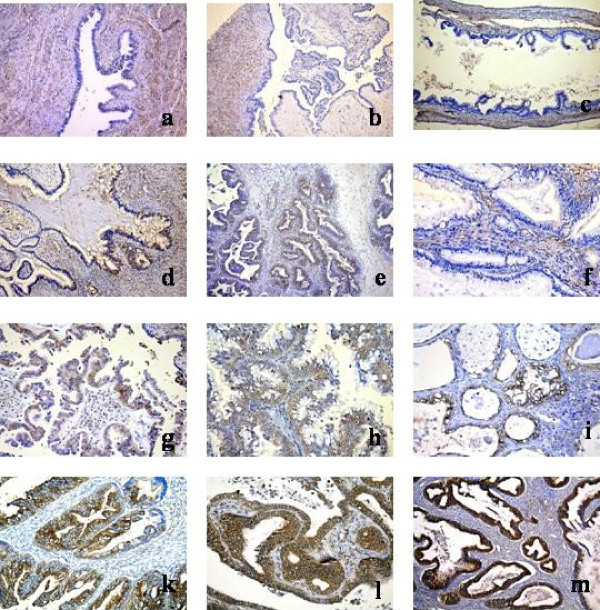
**Immunohistochemical analysis of YKL-40 protein expression in paraffin-sections of different types of ovarian tumor tissue (×40)**. A positive immunostaining appears as a cytoplasmic, granular brown-colored staining. a: Normal ovarium surface epithelium, no staining; b: borderline serous tumor, no staining; c: borderline mucinous tumor, no staining, d: borderline mucinous tumor, 30% positivity; e: well-differentiated serous adenocarcinoma, 50% positivity; f: well-differentiated mucinous adenocarcinom, no staining; g: moderately differentiated serous adenocarcinoma, 30% positivity; h: moderately differentiated mucinous adenocarcinoma, 40% positivity; i: moderately differentiated clear cell carcinoma, 40% positivity; k: moderately differentiated endometroid adenocarcinoma, 70% positivity; l: moderately differentiated serous adenocarcinoma, 70% positivity; and m: moderately differentiated endometroid adenocarcinoma, 80% positivity.

The distribution of the YKL-40 score in the borderline ovarian tumors was: level 1 (negative): 44 (24%); level 2: 74 (41%); level 3: 35 (19%) and level 4: 28 (16%). The distribution of the YKL-40 score in the OC samples was: level 1 (negative): 112 (24%); level 2: 101 (21%); level 3: 138 (29%) and level 4: 122 (26%). Figure 1 illustrates representative examples of the different levels of YKL-40 staining in borderline ovarian tumors and OC. The cancer cells showed a diffuse granular staining of the cytoplasm. Staining of membranes and nuclei were not observed. Borderline ovarian tumors appeared with the same staining pattern as the carcinomas, but with reduced staining intensity (Figure 1, b–d). YKL-40 protein expression was found in all the different histological types of OC (Figure 1). The YKL-40 protein was also expressed in inflammatory cells such as macrophages, mast cells and neutrophils. These results have been confirmed by double-labeling methods verifying co-expression of YKL-40 and tryptase (mast cells) and CD68 (macrophages), respectively (data not shown).

The association between clinico-pathological variables and YKL-40 score is shown in Table 1 and Table 2. The YKL-40 protein expression (staining percentage scale) in OC was associated with FIGO stage (p < 0.0001) and histological type of tumor (p < 0.0001), and the YKL-40 expression (staining percentage scale) was higher in the OC than in the borderline tumors (p = 0.0019). Also a tendency towards a correlation between reduced YKL-40 protein expression and radicality of surgery was observed (p = 0.05).

**Table 1:** Clinical characteristics and YKL-40 expression in tumor tissue and plasma YKL-40 levels from patients diagnosed with borderline ovarian tumor#

Characteristic		YKL-40 protein expression (N = 181)		Plasma YKL-40 (μg/l) (N = 19)
	N (%)	Median (range)	N (%)	Median (range)
**FIGO stage:**				
I	160 (88)	5 (0–90)	19 (100)	42 (20–180)
II	5 (3)	5 (0–30)		
III	16 (9)	7.5 (0–30)		

**Histological type of tumor:**				
Cystadenoma (NOS), borderline	14 (8)	7.5 (5–20)	2 (10)	39 (38–40)
Serous cystadenoma, borderline	95 (52)	5 (0–80)	6 (32)	42 (31–142)
Mucinous cystadenoma, borderline	69 (38)	5 (0–90)	11 (58)	37 (20–180)
Endometroid cystadenoma, borderline	3 (2)	5 (5–40)		

# Scoring of YKL-40 expression was based on the proportion of cells in a given tumor specimen exhibiting distinct cytoplasmic immunopositivity as well as intensity of staining percentage scale: 0, 5, 10, 20, 30, 40, 50, 60, 70, 80, 90, and 100. The YKL-40 scoring results was transformed into a four-tiered scale: 1 = negative; 2 = 5% positive tumor cells; 3 = 10% positive tumor cells and 4 = 20% or more positive tumor cells.

**Table 2:** Clinical characteristics and YKL-40 expression in tumor tissue and plasma YKL-40 levels from patients diagnosed with ovarian cancer#

Characteristic		YKL-40 protein expression (N = 473)		Plasma YKL-40 (μg/l) (N = 76)
	N (%)	Median (range)	N (%)	Median (range)
**FIGO stage:**				
I	157 (33)	10 (0–90)	17 (22)	59 (20–340)
II	48 (10)	5 (0–30)	4 (5)	53 (39–259)
III	245 (52)	10 (0–70)	47 (62)	168 (32–1808)
IV	23 (5)	20 (5–40)	8 (11)	320 (86–2650)

**Histological type of tumor:**				
Undifferentiated carcinoma	9 (2)	20 (0–40)	3 (4)	576 (156–1808)
Adenocarcinoma (NOS)	23 (5)	10 (0–30)	5 (6)	452 (237–2650)
Serous adenocarcinoma	249 (53)	5 (0–70)	50 (66)	112 (20–548)
Mucinous adenocarcinoma	53 (11)	10 (0–80)	7 (9)	102 (20–856)
Endometroid adenocarcinoma	69 (15)	10 (0–90)	8 (11)	93 (29–193)
Clear-cell neoplasm	46 (10)	10 (0–40)	2 (3)	220 (59–380)
No information	-	-	1 (1)	340

**Histological grade of tumor:**				
Grade 1	124 (26)	10 (0–60)	17 (22)	61 (20–356)
Grade 2	165 (35)	10 (0–80)	27 (36)	162 (32–856)
Grade 3	183 (39)	10 (0–90)	26 (34)	144 (20–2650)
No information	-	-	6 (8)	360 (29–1808)

# Scoring for YKL-40 expression was based on the proportion of cells in a given tumor specimen exhibiting distinct cytoplasmic immunopositivity as well as intensity of staining percentage scale: 0, 5, 10, 20, 30, 40, 50, 60, 70, 80, 90, and 100. The YKL-40 scoring results was transformed into a four-tiered scale: 1 = negative; 2 = 5% positive tumor cells; 3 = 10% positive tumor cells and 4 = 20% or more positive tumor cells.

The highest frequency of any level of positivity of YKL-40 protein expression was observed in serous adenocarcinomas, where 123 out of 249 tissues were scored positive (49%).

### Preoperative plasma YKL-40 in patients with borderline ovarian tumors and in OC

Plasma samples for YKL-40 analysis were available from 19 patients with borderline ovarian tumors (median age 45 years, range 38 – 76 years) and from 76 patients with OC (median age 63 years, range 37 – 79 years). The median preoperative plasma level of YKL-40 in the OC patients was 125 μg/l (range 20 – 2650 μg/l) and significantly higher than the level in 144 healthy women (median 32 μg/l, range 20 – 143 μg/l; p < 0.001). 67% (51/76) of the OC patients had a plasma YKL-40 level above the age-adjusted 95^th ^percentile of the healthy women. Patients with borderline ovarian tumors did not have elevated plasma YKL-40 (median 42 μg/l, range 20 – 180 μg/l) compared to healthy women. 21% (4/19) of the patients with borderline tumors had a plasma YKL-40 level above the age-adjusted 95^th ^percentile of the healthy women. Table 1 and Table 2 gives the relationship between preoperative plasma YKL-40 and standard clinical-pathological variables. Plasma YKL-40 increased with increasing FIGO stage among OC patients (p = 0.0001). Furthermore, plasma YKL-40 was correlated with serum CA-125 (Spearmans rho = 0.47, p < 0.0001) and age (rho = 0.59, p < 0.0001). No correlation was found between plasma YKL-40 and the YKL-40 tissue expression percentage score (rho = 0.032, p = 0.081).

### YKL-40 expression in OC and prognosis

**Figure 2:**
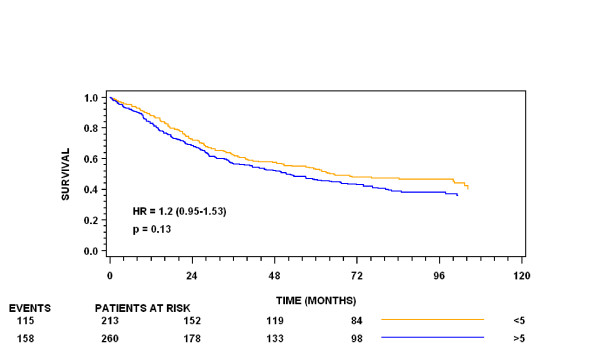
**Kaplan-Meier survival curves showing the association between YKL-40 protein expression and overall survival**. YKL-40 is dichotomized in cellular percentage score in two groups (< 5% vs. ≥ 5% positivity og the cancer cells).

Univariate survival analysis demonstrated that YKL-40 tissue expression (negative or ≥ 5% positive tumor cells) was not associated with survival (HR = 1.2; 95% CI: 0.95 – 1.53) (Figure 2). Similarly, using the four-tiered scale YKL-40 tissue expression was confirmed of no evidence of separation (data not shown). Including YKL-40 tissue expression (negative or ≥ 5% positive tumor cells) in a multivariate Cox regression analysis with FIGO stage, patient age, radicality of surgery and serum CA-125 as other parameters, showed no prognostic impact of YKL-40 tissue expression (HR = 0.97; 95% CI: 0.99 – 1.01) (data not shown).

### Plasma YKL-40 and prognosis

**Figure 3:**
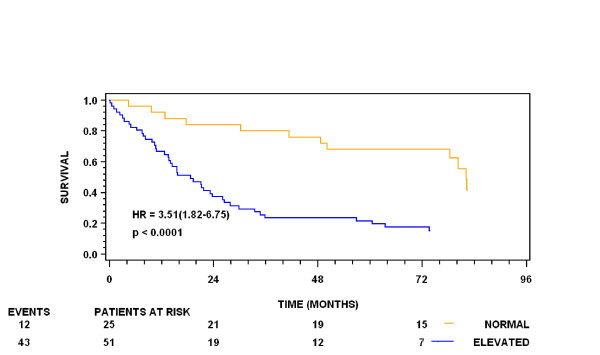
**Kaplan-Meier survival curves showing the association between preoperative plasma YKL-40 and overall survival**. Plasma YKL-40 is dichotomized in normal vs. elevated plasma YKL-40 levels. The cut-off point is the age-adjusted plasma YKL-40 value corresponding to the 95^th ^percentile in healthy controls. The P-value refers to the log-rank test for equality of strata.

During follow-up 55 (72%) of the 76 OC patients died. Univariate Cox analysis of plasma YKL-40 (log transformed and treated as a continuous covariate) showed significant association with overall survival (HR = 2.85, 95% CI: 2.04 – 3.97, p < 0.0001). Univariate Cox analysis of plasma YKL-40 (dichotomized as normal vs. high age-corrected plasma YKL-40 level) showed also significant association with overall survival (HR = 3.51, 95% CI: 1.82 – 6.75, p < 0.0001). Figure 3 illustrates the Kaplan-Meier estimates of survival stratified by baseline plasma YKL-40 dichotomized as normal vs. high age-corrected plasma YKL-40 level.

Performing a multivariate Cox analysis including plasma YKL-40 (log transformed and treated as a continuous covariate), serum CA-125 (log transformed and treated as a continuous covariate), FIGO stage (I-IV), residual tumor after surgery (yes or no), and age showed that only plasma YKL-40 (HR = 2.13, 95% CI: 1.40 – 3.25, p = 0.0004) was found of independent prognostic value of overall survival. Age at surgery (HR = 1.02, 95% CI: 0.99 – 1.06), radicality after primary surgery (HR = 1.20, 95% CI: 0.44 – 3.33), serum CA-125 (HR = 1.08, 95% CI: 0.94 – 1.23) and FIGO stage (p = 0.63, FIGO 1 vs 4: HR = 0.61, 95% CI: 0.11 – 3.34, FIGO 2 vs 4: HR = 0.25, 95% CI: 0.03–2.37, FIGO 3 vs 4: HR = 0.66, 95% CI: 0.28–1.58) were found of no independent prognostic value in OC patients.

## Discussion

In the present study we found YKL-40 protein expression in OC cells with a higher expression score in OC than in borderline tumors, and a clear association with FIGO stage and type of histology, but not with survival. We used tissue micro array with a tissue core diameter of 2.0 mm, but although this is comparatively large we do not know whether the degree of heterogeneity of YKL-40 protein expression in OC tissue could have influenced our results. This was, however, not the case in an immunohistochemical analysis of YKL-40 protein expression in tissue micro arrays of breast cancer [18]. Our present results of OC are in accordance with this study of breast cancer, where no association was found between YKL-40 score and prognosis [18]. However, this is in contrast to tumor samples from glioblastomas, where YKL-40 acted as a biomarker of genetic and histological subtypes, radiation therapeutic response and prognosis [11,12,14,29,30]. Pair-wise combinations of markers have identified epidermal growth factor receptor variant III (EGFRvIII) and YKL-40 as prognostically important in patients with glioblastoma, providing patients with EGFRvIII-negative/YKL-40-negative tumours with the best prognosis [29].

There are no published studies on YKL-40 protein expression in OC tissue, and to our knowledge no publications exist on a close association between serum YKL-40, serum CA-125 and CA15-3 in patients with OC [23-26]. It has previously been shown that preoperative serum or plasma concentrations of YKL-40 are elevated in 65% of patients with FIGO stage I and II, and in 74% to 91% of patients with FIGO stage III and IV [23,24]. In addition, patients with high serum or plasma levels of YKL-40 with early-stage OC [24], stage III [23], or recurrent of OC [25] had shorter survival than OC patients with normal levels of YKL-40.

In the present study plasma was available from a small subgroup of patients with borderline ovarian tumors and OCs, and in accordance with the studies mentioned above [23-26] we also found that elevated preoperative plasma concentrations of YKL-40 significantly predicted short survival in patients with OC. However, we did not find any correlation between plasma and tissue score YKL-40 in corresponding tissue and YKL-40 level in plasma samples in this subgroup of OC patients. Two persons simultaneously assessed the patterns of YKL-40 protein expression of each tumor sample and discussed each sample if result were not concordant. It is often the case that there is no correlation between protein expression in cancer tissue and circulating levels of the protein. The reason for this observation is possibly due to a considerable contribution to the plasma YKL-40 level from the many tumor-associated inflammatory cells, which also produce the protein [21,22]. Perhaps the YKL-40 production of inflammatory cells in tumor tissue are linked to or reflect a more malignant phenotype of the tumor, thus contributing to the elevated plasma YKL-40 levels observed in patients with a poor prognosis.

## Conclusion

In conclusion, this study has shown that YKL-40 is expressed in OC cells, and a high expression is associated with a high FIGO stage and histological type of tumor. In contrast to plasma YKL-40, high tumor cell expression of the protein was not associated with poor prognosis. This may be due to contribution to the plasma YKL-40 level from tumor-associated inflammatory cells, which also produce the protein.

## Competing interests

The authors declare that they have no competing interests.

## Authors' contributions

EH contributed to the conception of study design, data collection, method, statistical analysis, interpretation of the results and drafting of the final manuscript. SKK (Principal Investigator of the MALOVA study) contributed to the conception and design of study and data collection. CKH (Co-Investigator of the MALOVA study) contributed to the conception of study design, data collection and statistical analysis. JB (Co-Investigator of the MALOVA study) contributed to the conception of study design. JSJ contributed to the conception of method, interpretation of the results and drafting of the final manuscript. MR contributed to the conception of method used in the analyses of YKL-40 and interpretation of the results. LO-M. contributed by data collection and statistical evaluation. LHC contributed to the conception of immunohistochemical method and interpretation of the results. IJC contributed to the understanding of statistical analysis and interpretation of results. PAP contributed to the conception of method and the YKL-40 AAb.

All authors read and approved the final paper.

## Pre-publication history

The pre-publication history for this paper can be accessed here:

http://www.biomedcentral.com/1471-2407/9/8/prepub
